# Integration of maternal genome into the neonate genome through breast milk mRNA transcripts and reverse transcriptase

**DOI:** 10.1186/1742-4682-9-20

**Published:** 2012-06-07

**Authors:** M Kemal Irmak, Yesim Oztas, Emin Oztas

**Affiliations:** 1 High Council of Science, Gulhane Military Medical Academy, Ankara, Turkey; 2 Department of Medical Biochemistry, School of Medicine, Hacettepe University, Sihhiye, Ankara, Turkey; 3 Department of Histology and Embryology, School of Medicine, Gulhane Military Medical Academy, Ankara, Turkey

## Abstract

Human milk samples contain microvesicles similar to the retroviruses. These microvesicles contain mRNA transcripts and possess reverse transcriptase activity. They contain about 14,000 transcripts representing the milk transcriptome. Microvesicles are also enriched with proteins related to “caveolar-mediated endocytosis signaling” pathway. It has recently been reported that microvesicles could be transferred to other cells by endocytosis and their RNA content can be translated and be functional in their new location. A significant percentage of the mammalian genome appears to be the product of reverse transcription, containing sequences whose characteristics point to RNA as a template precursor. These are mobile elements that move by way of transposition and are called retrotransposons. We thought that retrotransposons may stem from about 14,000 transcriptome of breast milk microvesicles, and reviewed the literature.

The enhanced acceptance of maternal allografts in children who were breast-fed and tolerance to the maternal MHC antigens after breastfeeding may stem from RNAs of the breast milk microvesicles that can be taken up by the breastfed infant and receiving maternal genomic information. We conclude that milk microvesicles may transfer genetic signals from mother to neonate during breastfeeding. Moreover, transfer of wild type RNA from a healthy wet-nurse to the suckling neonate through the milk microvesicles and its subsequent reverse transcription and integration into the neonate genome could result in permanent correction of the clinical manifestations in genetic diseases.

## Introduction

In the 1970s, human milk samples were shown to contain particles that exhibit many of the features characteristic of retroviruses (see Ref [1] for details of retroviruses). In particular, these human particles have a density in sucrose of 1.16-1.19 g/ml and contain a single-stranded 60 and 70 S RNA physically associated with a reverse transcriptase [2-17]. However, labeled cDNA prepared from these particles hybridized exclusively with human genomic DNA but not with mouse and cat DNA indicating a human origin for the particles [18]. The etiological role of retroviruses in mammary cancer of experimental animals coupled with observations of morphologically similar particles in human milk has motivated considerable interest in the biological role of these virions in human breast cancer. However, no correlation could be demonstrated between the presence of retrovirus-like particles in human milk samples and human breast cancer [19,20]. Thus, lacking formal proof of a human mammary tumor virus, the possibility that human breast cancer might also be intimately associated with oncogenic viruses faded in the 1980s [21]. An explanation for this discrepancy could be that those retrovirus-like particles were not virions, but some other kind of particle.

### Milk fat globules and microvesicles

**Figure 1:**
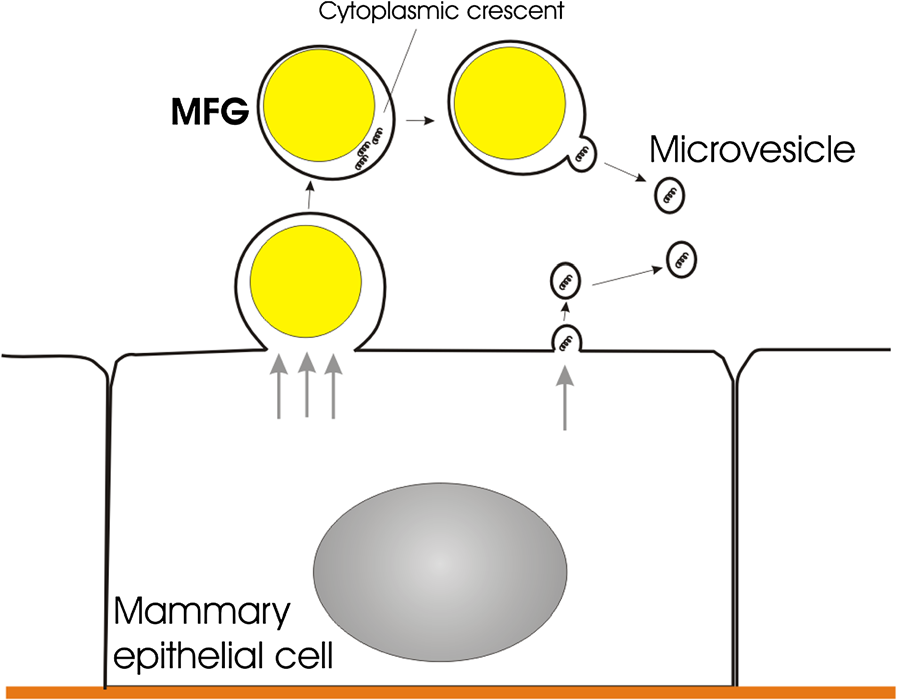
**Formation of breast milk microvesicles.** Breast milk microvesicles form directly from the apical parts of the mammary epithelial cells by an apocrine mechanism or indirectly from the cytoplasmic crescents of milk fat globules (MFG) by shedding, budding or blebbing, similar to the mechanism by which enveloped viruses are secreted from the cells.

The answer to the question about the nature of the retrovirus-like particles in human breast milk has come at the beginning of the 21st century. Breast milk has been found to contain microvesicles with a density in sucrose of 1.10-1.19 g/ml [22] comparable with previously identified retrovirus-like particles. In addition to biochemical and structural similarity, breast milk microvesicles also contain RNA and reverse transcriptase activity [23] as in retrovirus-like particles (see Ref [1] for details of microvesicles). Moreover, RNA of the breast milk microvesicles were demonstrated to be taken up by other cells supporting the notion that microvesicles could deliver RNA from cells of the mother, to cells in the offspring [24]. These microvesicles have been called exosome, lactosome or shedding microvesicles by the reporters but with no reference to those articles about the retrovirus-like particles in human milk published in 1970s. It is apparent that retrovirus-like particles of 1970s are identical with the microvesicles found more recently. Breast milk microvesicles form directly from the apical parts of the mammary epithelial cells by an apocrine secretion mechanism or indirectly from the cytoplasmic crescents of milk fat globules (MFG) by shedding, budding or blebbing (Figure 1), similar to the mechanism by which enveloped viruses are secreted from the cells [25-29].

Milk fat globules are triacylglycerol carriers of about 4 micrometer diameter secreted by the mammary epithelial cells and are the main source of energy for the infant [25]. Milk fat globules arise by the fusion of lipid droplets and are thus enveloped in a monolayer from the cytoplasmic leaflet of the endoplasmic reticulum (ER) membrane and are transported to the cell surface where they are pinched off into the alveolar space entirely surrounded in a layer of plasma membrane [30,31]. Variable amounts of cytoplasm are often entrained between the inner monolayer and the outer bilayer (Figure 1). These are generally called cytoplasmic crescents [32]. Cytoplasmic crescents contain nearly all intracellular membranes and organelles of the milk-secreting cell, except nuclei, and they represent an important route of cellular substances into milk, such as mRNAs and proteins [33]. About 100 proteins were identified in the membrane and cytoplasmic crescents of milk fat globules [34]. While these proteins have a very low nutritional value, they play important roles in various cell processes such as vesicle trafficking, cell signaling, protein synthesis, binding, folding, intracellular transport, antigen presentation (MHC class I and II molecules), receptor activity and immune functions [35,36]. The vesicle trafficking proteins identified include ADP ribosylation factor-1 (Arf1) [22], Rab1 and SNARE proteins [37]. Proteins such as clusterin, CD55 and CD59 protect microvesicles against complement lysis [38]. Among the identified proteins involved in folding and protein destination are cyclophilin A, and heat shock proteins [39]. Proteomes of MFG and microvesicle fractions mostly share the similar proteins, but microvesicles are especially enriched with proteins related to “caveolar-mediated endocytosis signaling” pathway [25]. In addition to this rich protein content, cytoplasmic crescents of MFGs were demonstrated to contain substantial quantities of high-quality RNA; about 14,000 transcripts representing the MFG transcriptome [40]. The top networks most highly associated with the MFG gene list were *1*) cellular function and maintenance cell signaling, and nucleic acid metabolism; *2*) cell cycle, *3*) DNA replication, recombination, and repair, *4*) protein synthesis, gene expression, and RNA trafficking [40]. It is plausible to suggest that transcriptome of cytoplasmic crescents reflects the RNA content of breast milk microvesicles which are capable of reverse transcription of the RNA into double-stranded DNA with their reverse transcriptase activity.

### Retrotransposons

A significant percentage of the mammalian genome appears to be the product of reverse transcription, containing sequences whose characteristics point to RNA as a template precursor [41]. These are mobile elements that move by way of transposition and are called retrotransposons [42]. They can be grouped into two large classes:

a. Retroviral-like retrotransposons. They resemble retroviruses, but lack a protein coat. They move themselves in and out of chromosomes by a mechanism that is identical to that used by retroviruses. These elements are present in organisms as diverse as yeast, flies, and mammals; unlike viruses they have no intrinsic ability to leave their resident cell but are passed along to all descendants of that cell through the normal process of DNA replication and cell division [42]. The first step in their transposition is the transcription of the entire transposon, producing an RNA copy of the element that is typically several thousand nucleotides long. This transcript, which is translated as a messenger RNA by the host cell, encodes a reverse transcriptase enzyme. This enzyme makes a double-strand DNA copy of the RNA molecule *via* an RNA/DNA hybrid intermediate, precisely mirroring the early stages of infection by a retrovirus. Like retroviruses, the linear double-stranded DNA molecule then integrates into a site on the chromosome by using an integrase enzyme that is also encoded by the element [42].

b. Nonretroviral retrotransposons. A large fraction of the human genome (about 40%) is composed of nonretroviral retrotransposons [43]. They move *via* a distinct mechanism that requires a complex of an endonuclease and a reverse transcriptase. The RNA and reverse transcriptase have a much more direct role in the recombination event than they do in the retroviral-like retrotransposons. RNA copy of the element is central to the incorporation of the element into the target DNA, acting as a direct template for a DNA target-primed reverse transcription event [42]. Basically, an endonuclease - reverse transcriptase enzyme complex attached to the RNA of the retrotransposon nicks the target DNA at the point at which insertion will occur. This cleavage releases a 3’-OH DNA end in the target DNA, which is then used as a primer for the reverse transcription step. This generates a single-stranded DNA copy of the element that is directly linked to the target DNA. In subsequent reactions, further processing of the single-stranded DNA copy results in the generation of a new double-stranded copy of the nonretroviral retrotransposon that is inserted at the site of the initial nick [42]. Nonretroviral retrotransposons are the major constituents of our genome, and the wide diversity of retrotransposons compared to the limited diversity of retroviruses suggests that most retrotransposons stem from RNA of other sources, maybe from about 14,000 transcriptome of breast milk microvesicles. Microvesicles of breast milk seem to be the appropriate structures for housing and delivering genes. But, after entering the body of a neonate, how can they penetrate mucus layers, move through the bloodstream, and transfer their RNA into the cells of the infant?

### Transport of breast milk microvesicles in the neonate

Viruses provide information that can be used by microvesicles to deliver their RNAs in the neonate [44]. Lactogenic transmission plays an important role in the biology of viruses, for example about one third of mother to child HIV infections are attributed to lactogenic infections [45]. Glycoproteins on the viral envelope provide protection from proteolytic enzymes and low pH in the stomach of the infant [41]. The great permeability of the gut of newborn facilitates the entry of the virus *via* ingestion of infected milk [46]. Viruses pass from intestinal epithelial cells through transcytosis and then, *via* the lymphatic system, into the systemic circulation [47-50]. Transcytosis is the process by which macromolecules internalized within caveolae are transported from the apical side of polarized cells to the basal side [44,51,52]. Transcytosis of viruses occurs widely in many polarized epithelial cell types after caveolar endocytosis. This process is rapid and viruses transcytose from apical to basolateral of the epithelial cells without infection [53,54]. Thus, caveolar endocytosis overcomes the epithelial and endothelial barriers by means of transcytosis, thereby delivering the viruses from the intestine to the tissues of the neonate. Extensive glycosylation of the viral envelope proteins renders them nearly invisible to immunoreactive cells and neutralizing antibodies in the circulation [41].

Like viruses, microvesicles are also resistant to degradation in the stomach of milk-fed infants and maintain their structure and function even at low pH and in the presence of the proteolytic enzyme pepsin [22,55,56]. Intestinal epithelial cells have been shown to secrete microvesicles from their basolateral side [57] and serum contains microvesicles originated from the gut epithelium [58,59]. With their molecular machinery for caveolar endocytosis and transcytosis, breast milk microvesicles could also be released at the basolateral surface of enterocytes passing into the systemic circulation of the infant. The presence of clusterin, CD55 and CD59 makes microvesicles resistant to complement lysis, and like viruses, microvesicles seem to be sufficiently stable to survive in the extracellular environment [60].

### Mechanisms of the microvesicle endocytosis by target cells

Viruses are valuable models of cellular entry and intracellular trafficking pathways. Membrane-bound compartments newly formed from the host cell surface normally enter the endosomal/lysosomal network, which is an inhospitable environment [61]. Therefore, after cellular uptake, microvesicles must harbor a mechanism that mimics that used by viral particles to escape from the endocytic/lysosomal pathway and proceed to the nucleus [62]. Caveolar internalization route mediates trafficking of viruses, such as Simian virus 40 (SV40), to the endoplasmic reticulum (ER), thus avoiding degradation in lysosomes [44]. Most of the viruses that enter cells *via* caveolar endocytosis are nonenveloped and are less than 55 nm. Evidently, the actual size of a single caveolae is very small (60–80 nm) to allow the accommodation of these viruses [44]. Caveolae pinch off from the plasma membrane and deliver their contents either to ER or to the nucleus [63]. Thus, microvesicles containing the molecular machinery for caveolar endocytosis and with a diameter of 50 nm must be internalized *via* caveolar endocytosis [64]. As a result, this pathway provides a direct route to deliver the microvesicular RNA from the plasma membrane to the ER or nucleus. Several other non-enveloped viruses also use caveolar endocytosis, including the ECHO 1 virus and coxsackie B; but in contrast to SV40, the endoplasmic reticulum is not involved in further steps of their intracellular transport [65,66]. This indicates that traffic to the ER is an active process mediated by the viruses. Therefore, microvesicles must contain a molecular machinery for vesicle trafficking and fusion and also must be capable to translocate these molecules to the outer surface of the vacuolar membrane as demonstrated in Legionella pneumophila (see Ref [1] for details of SV40 and Legionella pneumophila). Legionella pneumophila can manipulate host cell vesicular trafficking pathways and establish a vacuole, delivered directly from caveolae to the endoplasmic reticulum thus making it a model for milk microvesicles. Cells that do not develop caveolar invaginations have caveolar-equivalent plasma membrane domains, so-called "lipid rafts." Lipid-raft-dependent but caveolae-independent internalization pathways [67,68] also support the entry of some viruses including picornaviruses, papillomaviruses, filoviruses and retroviruses.

### Breast milk microvesicles as gene delivery vehicles

We suggest here that transfer of maternal mRNA to the suckling neonate through the milk microvesicles and its subsequent reverse transcription and integration into the neonate genome may form the basis of the presence of retrotransposons in the neonate. Moreover, the enhanced acceptance of maternal allografts in children who were breast-fed [69-71] and tolerance to the maternal MHC antigens after breastfeeding [72] may stem from RNAs of the breast milk microvesicles that can be taken up by the breastfed infant and receiving maternal genomic information. The above considerations may also form the basis of neonatal gene therapy *via* breast milk.

### Advantages of gene therapy in infancy

Gene therapy has become an invaluable tool to explore potential therapeutic applications to various acquired or inherited diseases (see Ref [1] for details of current gene delivery systems). However, immune responses to the therapeutic protein pose a significant hurdle for successful gene therapy. Problematic immune responses can include the development of a cytotoxic T lymphocyte response that results in the destruction of genetically-modified cells and/or the formation of antibodies directed against the therapeutic protein [73]. One approach to avoid an immune response is to perform gene therapy in newborns, which takes advantage of the fact that the immune system is relatively immature at birth. Mature T cells are not present during early infancy and the antibody repertoire is not fully established for many months. IgG antibodies to protein antigens are formed in early infancy, but IgG antibodies to polysaccharides do not appear until 2–2.5 years of age [74]. Newborns also have low serum complement levels [75]. When an antigen is introduced into immunologically immature newborns, they may, upon reaching maturity, become unresponsive to immunization with that antigen (neonatal tolerance). This immunological tolerance is characterized by the absence of both antibody and cell-mediated responses, and it is specific for the original antigen [74]. High antigen levels are more efficient at inducing tolerance. Reactive lymphocyte clones may be inactivated or deleted by exposure to these macromolecules during the early stages of maturation. Gene therapy that is initiated before the maturation of the immune system may thus limit the adverse immune response and thereby lengthen the duration of transgene expression. Application of gene therapy to treat genetic diseases has additional advantages when performed in newborns. Because of the minimal adverse effect of the underlying disease on cells of the newborn, the relatively small size of infants which makes the logistics of performing gene therapy simpler, and the large amount of future growth, gene therapy may be more successful in newborns than in older children or adults [76]. Many metabolic disorders could be treated by gene therapy during the neonatal period if prenatal diagnoses are made and the appropriate regulatory requirements have been met.

### Gene therapy through wet-nursing

In spite of continuous technological progress in gene therapy, most clinical results have been disappointing even in the applications performed during neonatal period. The reasons for this are many and include difficulty targeting the appropriate organ and low level expression of the therapeutic gene product [77]. We thought that many of these difficulties may be avoidable by applying the gene therapy in neonates through wet-nursing. Before the invention of bottles and commercial formula, wet-nursing “breastfeeding another’s baby” was the safest and most common alternative to the natural mother’s breast-milk [78]. Although this method is becoming less fashionable, there are still families who use this feeding method [78]. Despite the possibility of biological mother to have the similar genetic disorder as in neonate, in a gene therapy application through wet-nursing, we expect that a healthy wet-nurse would not carry the mutant gene. Therefore, gene therapy through wet-nursing gives permanent gene transfer and it would give a first option to parents following prenatal diagnosis of inherited disease, where the current choices are termination of pregnancy or acceptance of an affected child. A gene therapy through wet-nursing would be extremely safe, reliable and effective at treating the genetic disease. In practice, an effective and comprehensive prenatal screening policy for the more common genetic disorders would need to be implemented and parents at risk of having an affected child would be seen early in the antenatal period for counseling and therapy as appropriate. The application of this method in humans will critically depend on our ability to demonstrate its safety and efficiency in preventing or treating genetic diseases. Improved knowledge of the candidate diseases to be treated is also vital. To improve this simple process of milk donation, parents, as well as the community, need education. In this method, milk donation should start after taking a detailed medical history of the donor mother with special attention to infectious diseases and having the donor undergo screening tests for HIV, Hepatitis B and C, HTLV-I, CMV, and syphilis. One of the practical problems that must be addressed is the length of time taken for the screening tests. Therefore, attempts to find a donor mother should start as soon as possible, after a genetic disorder is diagnosed in prenatal period [79]. It remains to be seen whether this method will provide better treatments for genetic diseases than those that currently exist [80].

Following considerations should also be kept in mind when planning neonatal gene therapy through wet-nursing in future studies:

a. Pasteurization does not eliminate the presence of nucleic acids from human milk, but it affects the quality of the nucleic acids present. Pasteurized human milk samples from milk banks are therefore less useful in milk-based gene therapy studies [81].

b. Gene therapy raises the possibility of introducing genetic modifications into the recipient’s germ cells, which could then be passed on to future generations. Studies suggest a low risk of germ line transmission [82].

c. By wet-nursing, a kind of relativity is established between the breastfed infant and the offspring of the wet-nurse.

### A scenario for the natural gene therapy through wet-nursing

**Figure 2:**
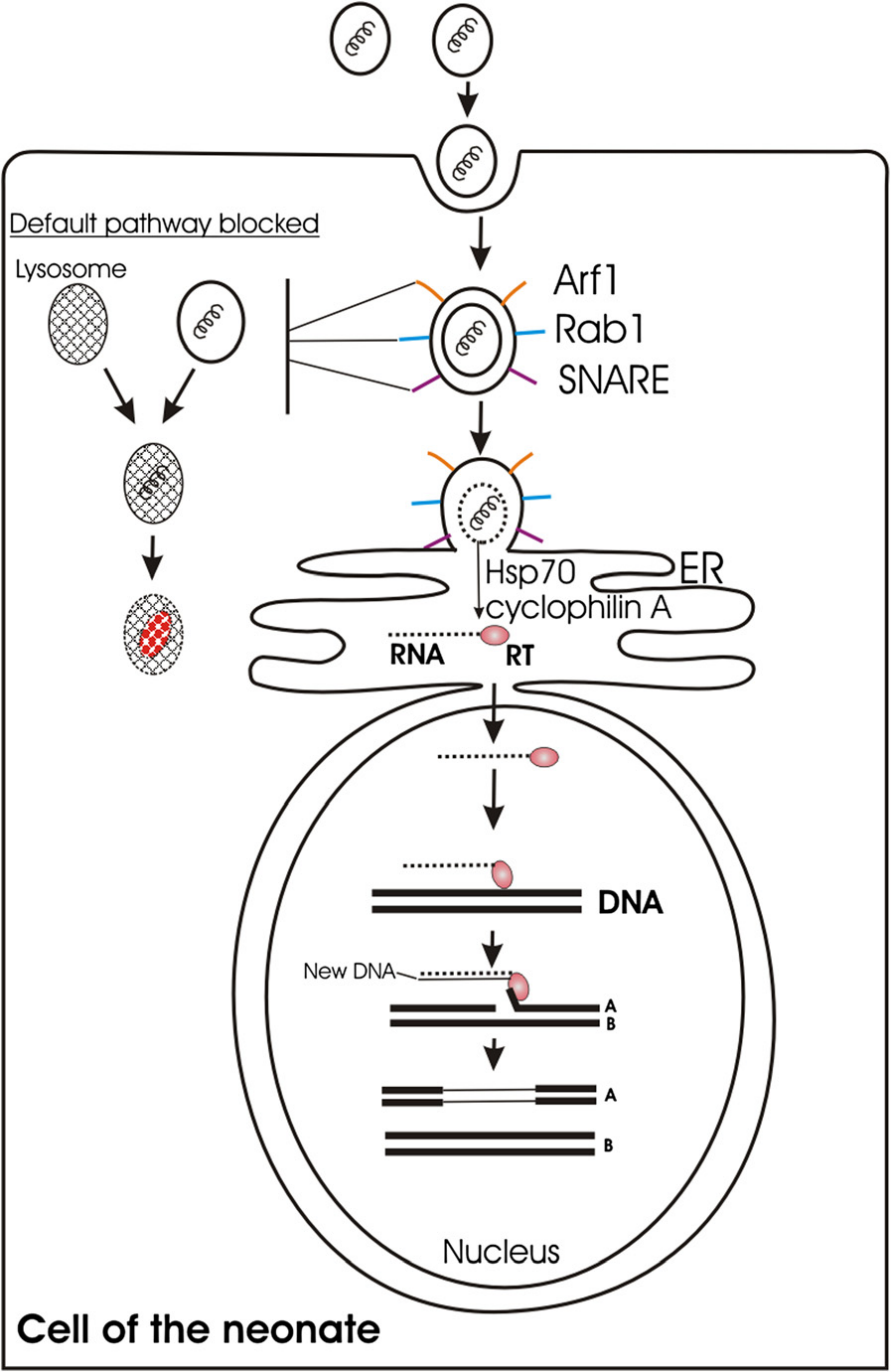
**Fate of breast milk microvesicles after endocytosis by target cells.** After caveolar endocytosis, microvesicles translocate Arf1, Rab1 and SNARE proteins to the outer surface of the vacuolar membrane by a syringe-like mechanism. These proteins help the microvesicles to pass from caveola to the endoplasmic reticulum and fusion of the vacuoles with ER. In ER, decoating of the microvesicular membrane occurs with the help of Hsp 70 and cyclophilin A and the released RNA is translocated into the nucleus directly together with reverse transcriptase (RT). In the nucleus, the linear copy of the microvesicular RNA is inserted into chromosomal DNA with the aid of cellular endonuclease and transcribed into a double-stranded DNA by the microvesicular RT. New DNA achieves the status of a cellular gene and replicated by cellular enzymes in concert with chromosomal DNA as in nonretroviral retrotransposons.

Mucopolysaccharidosis (MPS) type VII, caused by deficient activity of beta-glucuronidase, is a lysosomal storage disease and has multisystemic manifestations including organomegaly, and skeletal, neural, cardiovascular, and ocular abnormalities [83]. Neonates are essentially normal at birth because of the maternal enzymes that eliminate the substrate accumulation in the fetus during prenatal life. Neonatal gene therapy by wet-nursing in neonates with MPS VII begins at days 1–2 of life. Breast milk microvesicles containing wild type RNA tolerate the gastric environment of the infant, the pH of which is about 5 and decreases with time to reach adult levels (pH 1–3) at 2 years of age [22]. After trancytosis from the intestinal epithelial cells by caveolar endocytosis, microvesicles are not transported to any extent in the portal venous blood. Instead, they are collected by the lymphatic vessels of the abdominal region and pass to the systemic blood *via* the thoracic duct as in chylomicrons [84]. Immature nature of the immune system of the neonate coupled with the extensive glycosylation of the microvesicular membrane proteins renders them nearly invisible to immunoreactive cells and neutralizing antibodies in the circulation whereas the presence of clusterin, CD55 and CD59 protects microvesicles against complement lysis. Caveolar endocytosis also overcomes the endothelial barriers by means of transcytosis without any change, thereby delivering the microvesicles including RNAs of wet-nurse to the neonatal cells. After binding to the plasma membrane *via* MHC class I antigens, microvesicles enter the host cells through the caveolar endocytosis. For the cells that do not have caveolar invaginations, microvesicles use the lipid-raft-dependent internalization pathways. After penetration into the cell, microvesicles move along microtubules toward the ER. The traffic to the ER is an active process, and microvesicles containing molecular machinery for vesicle docking and fusion (Arf1, Rab1 and SNARE proteins) are able to translocate these molecules to the outer surface of the vacuolar membrane by a syringe-like mechanism. Arf1 and Rab1 proteins help the microvesicles to pass from caveola to the endoplasmic reticulum; whereas SNARE proteins take role for the fusion of the vacuoles with ER. In ER, decoating of the microvesicular membrane occurs with the help of molecular chaperones such as Hsp 70 and cyclophilin A and the released RNA is translocated into the nucleus directly together with reverse transcriptase. In the nucleus, the linear copy of the RNA is inserted into chromosomal DNA with the aid of cellular endonuclease and transcribed into a double-stranded DNA by the microvesicular reverse transcriptase (Figure 2). After integration with the neonate genome, new DNA achieves the status of a cellular gene and replicated by cellular enzymes in concert with chromosomal DNA as in nonretroviral retrotransposons. In this way, about 14,000 transcripts representing the microvesicular transcriptome from the wet-nurse can be expressed in the neonate treating almost all kinds of genetic diseases. Because of the wide networks associated with the microvesicular gene list including cellular function, cell signaling, nucleic acid metabolism; cell cycle, DNA replication, recombination, and repair; protein synthesis, gene expression, and RNA trafficking, this method is capable to treat a wide range of genetic disease. In conclusion, with the neonatal gene therapy through wet-nursing, the transfer of wild type mRNA to the suckling neonate through the milk microvesicles and its subsequent reverse transcription and integration into neonate genome result in the permanent correction or amelioration of the clinical manifestations of the genetic disease. Enhanced rate of cell division at the time of neonatal period easily allows the integration of new genes and result in the amplification of the genetically modified cells. After therapy, infants have normal serum beta-glucuronidase enzyme activity and clinical signs of disease, such as cardiac abnormalities are absent or minimal. The neonates remain ambulatory, versus untreated affected infants, who are unable to stand or walk by the age of 2 years.

## Competing interests

The authors declared that they have no competing interest.

## Authors’ contributions

MKI reviewed the relevant literature and wrote the manuscript. YO proposed the concept of theory. EO was primarily responsible for finding related references and drawing the figures. All authors read and agreed the final manuscript.
